# The Dietary Inclusion of Ensiled Olive Cake Increases Unsaturated Lipids in Milk and Alters the Expression of Lipogenic Genes in Mammary and Adipose Tissue in Goats

**DOI:** 10.3390/ani13213418

**Published:** 2023-11-03

**Authors:** Marina C. Neofytou, Ariadne-Loukia Hager-Theodorides, Eleni Sfakianaki, Panagiotis Simitzis, Simoni Symeou, Dionysis Sparaggis, Ouranios Tzamaloukas, Despoina Miltiadou

**Affiliations:** 1 Department of Agricultural Sciences, Biotechnology and Food Science, Cyprus University of Technology, P.O. Box 50329 Limassol, Cyprus; maneofyt@gmail.com (M.C.N.); simonisymeou@hotmail.gr (S.S.); ouranios.tzamaloukas@cut.ac.cy (O.T.); despoina.miltiadou@cut.ac.cy (D.M.); 2 Laboratory of Animal Breeding and Husbandry, Department of Animal Science, Agricultural University of Athens, 11855 Athens, Greece; a.hager@aua.gr (A.-L.H.-T.); lenasfakianakis@hotmail.com (E.S.); 3 Agricultural Research Institute, P.O. Box 22016 Nicosia, Cyprus; dsparaggis@ari.moa.gov.cy

**Keywords:** ensiled olive cake, milk fatty acids, gene expression

## Abstract

**Simple Summary:**

Olive oil is known worldwide for its beneficial effects on human health. However, during its extraction, a great quantity of by-products is produced. Their utilization as a replacer of forage in goats could minimize the costs related to their management and result in their sustainable utilization as dietary ingredients. As shown in the present experiment, milk fat and protein percentages were gradually increased with increasing ensiled olive cake (OC) inclusion rates in the diets, while the milk yield was not affected. The concentration of milk mono-unsaturated fatty acids (MUFAs) was also enhanced, while OC dietary supplementation was associated with the increased expression of specific genes in the mammary and adipose tissues of goats. As can be concluded, OC inclusion of up to 20% (DM) in goats’ diets as forage is recommended since milk protein, fat, and MUFA contents are increased without adversely affecting milk yield.

**Abstract:**

This study aimed to evaluate the effect of the dietary inclusion of ensiled OC on milk yield, composition, fatty acid (FA) profile, and the expression of selected genes involved in lipid metabolism in the udder and adipose tissue of goats. Seventy-two Damascus dairy goats in mid-lactation were assigned randomly to three iso-nitrogenous and iso-energetic diets containing 0, 10, and 20% of ensiled OC as a replacement of forage (OC0, OC10, and OC20, respectively) for 42 days. During weeks 5 and 6 of the trial, dry matter intake, milk yield, milk composition, and FA profiles were recorded, while mammary and perirenal adipose tissue samples were also collected from six animals per treatment from the OC0 and OC20 groups for gene expression analysis. No significant differences were observed among groups concerning milk yield, 4% fat-corrected milk, fat, or protein yield (kg/d). In contrast, the milk fat percentage was gradually increased with increasing OC inclusion rates in the diets, while milk protein percentages were elevated in both OC groups but significantly only in the milk of the OC20 group. The content of FA between C4:0 to C16:0 was reduced, while mono-unsaturated FA (MUFA) concentration was enhanced in the goat milk of OC groups. The OC feeding treatment was associated with the increased mammary expression of *SLC2A1* (*p* < 0.05), *VLDLR* (*p* < 0.01), *FABP3* (*p* < 0.01), and elevated *SLC2A1* (*p* < 0.05) and *FASN* (*p* < 0.01) gene expression in the adipose tissue of goats fed the OC20 diet. Overall, OC can be used in goats’ diets as a forage replacement, at least in the inclusion rate of 20% DM, since this could increase the milk protein and fat percentage and enrich its content with beneficial for human health lipids without adversely affecting milk production traits.

## 1. Introduction

The use of feeds rich in unsaturated fatty acids (UFA) in dairy animals is a strategy for improving milk fat composition for human consumption [1,2,3]. Crude olive cake (OC), a by-product of olive oil extraction containing a mixture of skins, pulp, woody endocarp, and seeds, is available in appreciable quantities in the Mediterranean areas and has been tested as a ruminant feed in different forms (fresh, dried, destoned, pelleted, or ensiled) [4]. OC silage represents an alternative feedstuff that can be used as a forage substitute, which, due to its enriched oleic acid content, it can also improve the fatty acid (FA) profile of produced milk and cheese [5,6,7]. The application of OC to ruminant nutrition has been mainly examined in sheep diets using different types of processed OC, like dried [8,9,10], partly destoned fresh [11], or ensilaged OC [5,6]. In all these studies, OC significantly affected milk lipid content by decreasing saturated fatty acids (SFAs), increasing the mono-unsaturated (MUFA) and overall unsaturation index of milk and dairy products. Additionally, studies in cows have reported enhanced MUFA and conjugated linoleic acid (CLA) content in milk and related cheeses using dried [12] or ensiled [7] OC. Up to today, there have been three studies on goats that incorporated different forms of OC by-products in their experimental diets, investigating their effects on milk’s FA composition [13,14,15]. However, in these studies, OC was used in combination with other dietary additives and compared to control diets, and thus, their results showed contradictory effects on goat milk’s FA composition that cannot be attributed to the OC inclusion alone. For instance, the study by Acro-Perez et al. [14] included ensiled OC along with olive leaves adding up to 20% of the diet DM, and this experimental diet was supplemented also with 2% (on DM) sunflower oil, which was not included in the control diet. As a result, the milk FA profile of this diet had increased SFAs, decreased MUFAs, and unaffected poly-unsaturated FAs (PUFAs) but increased CLA content compared to the control, which contradicted the results of OC inclusions in sheep and cows. Moreover, Molina et al. [13] included crude OC in experimental diets along with several other ingredients not included or included in different quantities in the control diet, such as fava beans, barley grains, beet molasses, and sunflower meal. As a result, they found decreased SFAs and increased CLA content in the goat milk of the OC-supplemented groups compared to the control, while oleic acid and the total MUFA and PUFA contents remained unaffected. Furthermore, Marcos et al. [15] investigated an experimental diet that, apart from the OC (3.5%), also included 8% dried distillers’ grains with solubles (DDGS) and 8% dry citrus pulp (DM basis), reporting an overall increase in milk PUFAs, linoleic and CLA content, and reduced SFA levels compared to the controls, but these effects cannot be attributed solely to OC since DDGS inclusion also affects the FA profile [16,17]. Thus, it remains unclear which is the effect of OC alone on goat milk quality and production and whether ensiled OC can be useful as a forage substitute in goat diets.

The underlying mechanisms of the effect of OC dietary supplementation on milk FA in ruminants have only been studied in relation to rumen microbiota alteration, fermentation, and rumen-derived FA [18,19] in attempts to elucidate the overall effect on ovine milk composition. The investigation of the effects of dietary OC supplementation on the expression of genes involved in fat metabolism (in the mammary or other tissues) has attracted limited interest. Previous studies using different diets, or supplements, rich in plant or marine lipids on FA composition have examined how lipid metabolism was affected in the mammary and adipose tissues by these dietary treatments [20,21,22,23]. More specifically, these studies examined the effects of diet alteration on the expression of genes involved in major lipogenic pathways (de novo synthesis, FA uptake and transport, and FA desaturation), as well as their regulatory elements (e.g., transcription factors). Recently, we found limited effects on the mRNA abundance of candidate genes known to be involved in the mammary and adipose tissues’ lipid metabolism in cows when OC silage replaced forage at 10% of the diet DM [7]. Species’ specificities of lipid metabolism have been well established [2,20,24], and so far, no studies have examined the effect of any form of OC supplementation on lipogenic gene expression in goats.

This study aimed to examine the effects of the inclusion of ensiled OC as the sole addition for forage replacement in the diet of mid-lactation Damascus goats. We tested the effects of the addition of three different levels (0, 10, and 20% on diet DM) of ensiled OC on milk production traits and the milk FA content. Furthermore, to elucidate the molecular mechanisms involved in the observed effects on milk FA composition, we performed differential expression analysis of 10 genes involved in the FA synthesis (*ACACA*, *FASN*, and *G6PDH*), FA uptake and/or translocation (*VLDLR*, *LPL*, *SLC2A1*, *CD36*, and *FABP3*), FA desaturation (*SCD1*), and transcriptional regulation (*PPARγ*) of lipid metabolism in the mammary and adipose tissues of goats.

## 2. Materials and Methods

### 2.1. Animals, Management, and Experimental Diets

All experimental procedures with animals were conducted at the Agricultural Research Institute of Cyprus according to the regulations of the national legislation [25] and international guidelines [26] for animal experimentation and approved by the corresponding departmental committee of the Cyprus University of Technology. For the purposes of this study, no animals were euthanized.

Seventy-two mid-lactating Damascus goats (98 ± 5 days in milk) of third parity were randomly allocated into 9 pens of 8 animals with average (±SEM) milk yield values (2.81 ± 0.13 kg per head per day) and body weights (59.05 ± 1.28 kg). Three pens or 24 animals per treatment were then randomly assigned into 3 iso-energetic and iso-nitrogenous feeding regimes that contained 0, 10, or 20% on DM of ensiled OC replacing forage (the OC0, OC10, and OC20 treatments, respectively) with a constant concentrate participation ratio of 60% DM in the diets for all treatments (Table 1). OC’s preparation and ensiling were conducted according to the method described by Neofytou et al. [7]. Briefly, the OC was collected using a 3-stage oil mill and ensiled using a black polyethylene film (8 mm thick) to facilitate the fermentation process. The film covering the pile was stretched to expel the air, and soil was used to cover the edges of the film. The ensiled OC was fermented in silos for 3 to 4 mo before use.

All goats were group-fed the control diet for a 3-week adaptation period and then the experimental diets for the next 6 weeks. In each pen, the animals were group-fed the diets to 1.1 times their maintenance energy and milk production requirements [27]. All feed ingredients, apart from OC, were offered manually as a total mixed ration two times per day after morning (0430 h) and evening (1630 h) milking. The OC supplement was fed directly only after morning milking, before feeding, and was totally consumed within 10 to 20 min of distribution. Dry matter intake (DMI) and feed samples for chemical analysis were collected at weeks 5 and 6 of the trial. Water was offered ad libitum. The chemical composition of the three experimental diets is presented in Table 1. Dry matter (DM), ash, crude fat, crude protein, crude fiber, and minerals were determined as described by AOAC [28]. Acid detergent fiber (ADF) and amylase-neutral detergent fiber (aNDF) were measured according to the method of van Soest et al. [29].

### 2.2. Measurements, Sampling, and Analysis

The goats were kept indoors and were machine-milked (Fulwood, Shropshire, UK) twice daily (at 0430 h and 1630 h), and their milk yields were recorded electronically (AfiMilk model Afifree 155, SAE Afikim Kibbutz, Israel). Individual raw milk samples for the determination of the milk composition were collected at the end of weeks 5 and 6 from each goat during two consecutive milkings (morning and evening). Measurements for the total fat, protein, lactose, and solid non-fat (SNF) of the milk were determined using a Lactostar milk analyzer (model 3510, Funke Gerber, Germany) [28]. Lipid extraction from the milk was performed according to the rapid double centrifugation method described previously by Symeou et al. [5]. In short, 20 mL of milk was centrifuged at 17,800× *g* (4 °C) for 30 min. The resulting fat was removed and placed in new tubes. The fat was then allowed to melt for 20 min at room temperature. The samples obtained were then recentrifuged at 19,500× *g* for 20 min (room temperature), and 2 mg aliquots of the resulting lipids were transferred to new tubes and dispersed via shaking in 1 mL of n-hexane.

The crude fat of the feeds was extracted using the Soxtec method [28], followed by the method described by Neofytou et al. [7]. Briefly, 2 mL of hexane was added to the aluminum pots which contained the residue of the fat and removed to a new glass tube (previously weighed). The fat residue was dried under a smooth stream of nitrogen gas, and the tube was weighed again. Subsequently, 1 mL of hexane was added in order to further dissolve the residue, and a 20–25 mg aliquot of the crude fat was transferred into new tubes.

The fatty acid methyl esters (FAME) of the milk and feed lipids were prepared according to the ISO [30] method and performed using a GCMS-QP2010 Plus Gas Chromatography-Mass Spectrometer (Shimadzu, Duisburg, Germany) equipped with a 100 m × 0.25 mm × 0.2 μm column (Agilent CP-Sil 88 fused silica capillary column) with a 1:20 split ratio. The column was held for 4 min at 70 °C after injection, increased at 13 °C/min to 175 °C, and then held at that temperature for 27 min. The temperature was then raised to 215 °C at a ratio of 4 °C/min and held for 36 min. Helium gas was the carrier gas at a rate of 1 mL/min, while both the injector and interface were held at the temperature of 225 °C. The obtained chromatographs were analyzed using Shimadzu GCMS Postrun Solution software, and each peak was identified by comparison of its retention index and mass spectra to the standards available and the mass spectral NIST libraries quantitated by peak integration and expressed as a percentage of the total fat.

Milk atherogenicity index (AI) was determined using the formula proposed by Ulbricht and Southgate [31]: AI = (C12:0 + 4 × C14:0 + C16:0)/(ΣMUFA + ΣPUFA) and the desaturation index (DI) was determined using the formula suggested by Garnsworthy et al. [32]: DI = (C14:1 *cis*-9 × 100)/(C14:0 + C14:1 *cis*-9). Fat-corrected milk yield at 4% of fat content (FCM 4%) was estimated according to Mavrogenis and Papachristoforou [33] for Damascus goats: FCM 4% = milk yield × (0.411 + 0.0147 × fat%).

### 2.3. Tissue Sampling for Expression Analysis

At the end of the experiment (week 6), mammary and adipose tissue biopsies were obtained from 6 animals from the control group (OC0) and 6 animals from the OC20 group that were randomly selected, under anesthesia, according to the method described previously by Miltiadou et al. [34], and the procedure took place approximately six hours after morning milking and feeding. Briefly, the perirenal adipose tissue was sampled by a veterinarian following lateral laparotomy and puncture using ultrasonography for the appropriate biopsy site selection, avoiding large vessels and other organs. Udder biopsies were taken from either the left or the right gland. The biopsy site was carefully selected to avoid large subcutaneous blood vessels. The preparation of the site involved shaving and washing with dilute betadine solution followed by sanitizing with ethanol (70%). Goats were provided with intravenous xylazine before anesthetizing the biopsy site via subcutaneous injection with lidocaine hydrochloride. An incision was made (~0.5–1.0 cm) using a scalpel blade (size 22). A Bard Magnum core biopsy instrument (Bard Peripheral Vascular Inc., Tempe, AZ, USA) with the accompanying biopsy needle (MN1210, 12 gauge × 10 cm) was used. The mammary and perirenal adipose tissues were dissected immediately, snap-frozen in liquid nitrogen, and stored at −80 °C.

### 2.4. Applied Molecular Techniques 

Primers were designed using the Primer-BLAST tool on the National Centre for Biotechnology Information (NCBI) platform using mRNA (preferably choosing the reference, where available) sequences for each target or housekeeping gene (Table 2). The primers were designed to avoid genomic amplification, and either the cross exon–exon boundaries or each primer of a pair was designed at different exons.

Total RNA from the perirenal adipose tissue (25 mg) and mammary gland tissue (50 mg) was isolated using a NucleoZOL reagent (MACHEREY-NAGEL GmbH & Co. KG, Düren, Germany), according to the manufacturer’s instructions. In detail, tissue of either 25 or 50 mg weight was initially homogenized in 500 μL of the NucleoZOL reagent. The samples of adipose tissue were then centrifuged at 12,000× *g* for 5 min, and the supernatant, below the layer of fat, was removed into a new tube. Then, 200 μL of RNAse-Freewater was added to the samples, which were consequently vortexed and incubated for 15 min at room temperature and then centrifuged at 12,000× *g* for 15 min. Supernatants were mixed with isopropanol of equal volume, incubated for 10 min at room temperature, and then centrifuged at 12,000× *g* for 10 min. Pellets were washed twice with ethanol and centrifuged for 3 min at 8000× *g*. Finally, the obtained pellets were air-dried and diluted in 60 μL of RNAse-Free water, and the samples were stored for further analysis at −80 °C. RNA purity (260/280 and 260/230) and concentration (ng/μL) were assessed using a micro-volume UV spectrophotometer (Quawell Spectrophotometer 3000, Lab Supplies Scientific, San Jose, CA, USA), and the samples were accepted if the 260/280 and 260/230 ratios were above 1.8. The cDNA was synthesized from 1 μg of total RNA in reactions of 20 μL using the PrimeScript RT-PCR Reagent Kit (TAKARA Bio INC, Shiga, Japan) following the instructions of the manufacturer, and the samples were finally stored at −20 °C for further analysis.

The mRNA abundance of the 10 selected genes, shown in Table 2, was assessed via real-time reverse transcription quantification PCR (RT—qPCR). To minimize the possible variation in the starting material and the mRNA extraction and cDNA synthesis efficiency between samples, the mRNA abundance was normalized using the mean (geometric) of 3 reference genes (ubiquitously expressed transcription (UXT), ribosomal protein S9 (RPS9) and ribosomal protein S15 (RPS15)) [35].

Real-time qPCR reactions were performed in an ABI 7500 Real-Time PCR system (Applied Biosystems, ThermoFisher Scientific, Waltham, MA, USA) using FastGene IC Green 2x Universal Mix (Bioline, London, UK). Each 10 μL reaction contained 1.0 μL of cDNA (synthesized from c.a. 12.5 ng total RNA), 400 nM of forward and reverse primer, and an IC Green Master Mix of 5.0 μL. The real-time qPCR analyses of each gene were performed using cDNA from 6 biological replicates, along with the 3 technical replicates per biological replicate. The qPCR thermal protocol used was as follows: 1 cycle of 95 °C for a duration of 2 min, 40 cycles of 95 °C for 5 sec, 60 °C for 30 sec, and 95 °C for 30 sec. This procedure was followed by a melt-curve analysis to examine the specificity of the amplification. Real-time PCR runs with efficiencies between 86 and 110% were considered acceptable and further used for data analysis.

The obtained data were analyzed using 7500 Fast Software for the quantitation relative to the standard curve with SYBR green reagents (Version 2.3; Applied Biosystems). The threshold cycle (C_T_) values were obtained for each reaction using the automatic C_T_ option, in which the baseline start was calculated using the software, as well as the end values and the threshold in the amplification plot for a set of reactions. To perform the relative quantitation of the levels of mRNA for each gene, a set of standards, consisting of 5-point serial dilutions (more specifically, 1:1, 1:3, 1:3^2^, 1:3^3^, and 1:3^4^) of cDNA prepared from a pool of cDNA from different tissues (adipose and mammary) were used along with the samples at every run. The results of the standard reactions (using triplicates for each dilution point) were used to generate the relative standard curve for each examined gene. The corresponding standard curve was then identified as the best-fit regression line of C_T_ (dependent variable) on the log(Qty) (independent variable with values of log(1), log(1/3), log(1/3^2^), log(1/3^3^), and log(1/3^4^)) described using the regression line formula C_T_ = m [log (Qty)] + b, where m, b, and Qty are the slope, the y-intercept, and the Qty of the relative mRNA level of the gene (i.e., the quantity of the gene). The levels of relative mRNA for each gene and each sample were estimated as the mean Qty of three technical replicates, obtained using the mentioned regression formula and applying the C_T_ values obtained from the reactions. Normalized expression was calculated as the ratio of the gene mean Qty/geometric mean of reference genes’ mean Qty.

### 2.5. Statistical Analysis

The DMI, milk yield, milk content, and milk FA composition/FA yield per day data were collected at week 5 and week 6 and were subjected to repeated measures analysis for a completely randomized design using SAS PROC MIXED (SAS version 9.4, SAS Institute Inc., Cary, CA, USA). The model included the fixed effects, the experimental diet (D), time (T), and their interaction (D × T), and the random effect of goats (pen). Statistical significance was declared at *p* < 0.05, and *p*-values ≤ 0.10 but >0.05 were interpreted as trending towards significance. Data referring to the FA composition of feeds were analyzed using one-way ANOVA with 3 replications, respectively.

The normalized relative mRNA expression data were subjected to non-parametric (distribution-free) analysis since the data did not meet the assumptions of one-way ANOVA. More specifically, the assumption of normality was not viable. The NPAR1WAY procedure of SAS (SAS version 9.4, SAS Institute Inc., Cary, CA, USA) was used to perform the nonparametric tests. For each gene, the dependent variable was the normalized mRNA expression level, and the classification variable was the treatment group (12 biological replicates, 6 per treatment groups OC0 and OC20). Differences between the two group populations having the same shape (when testing whether the two samples were likely to derive from the same population) were based on Wilcoxon rank scores. The test is also known as the Kruskal–Wallis chi-square test, with 1 DF declared significant when *p* < 0.05.

## 3. Results

### 3.1. Fatty Acid Profile of OC Silage and Experimental Diets

Table 3 shows the FA composition of dietary treatments and OC silage used. Feeding goats with ensiled OC had notable effects on all FAs, with oleic acid (C18:1 *cis*-9) being the major FA found at concentrations of 35 and 37% in the OC10 and OC20 diets, respectively. The content of C16:0 and C18:2n-6 was also abundant, whereas C18:0 and C18:3n-3 were found in lower quantities in all treatments. Both OC diets contained significantly decreased amounts of palmitic, linoleic, and linolenic acid compared to the OC0 diet. In contrast, stearic acid increased as the OC proportion was elevated in the goats’ diets (*p* < 0.001).

### 3.2. Milk Yield and Composition

Table 4 shows the DMI, 4% fat-corrected milk, the daily yield of milk, fat, and protein, and the milk composition. OC inclusion did not influence intake, the yield of milk and protein, or the fat-corrected milk of the goats. Similarly, the lactose and SNF percentages did not differ between treatments. However, the content of milk fat increased significantly as OC rates were raised in the goats’ diets, and the milk fat yield tended to be elevated in the OC groups (*p* = 0.06). Additionally, the milk protein percentage was increased with OC’s inclusion in both the OC10 and OC20 treatments, though significantly only for the OC20 group compared to the control (*p* < 0.001).

### 3.3. Milk’s Fatty Acid Composition

The results of FA expressed as % and g/day determined in the milk of the OC0, OC10, and OC20 groups are shown in Table 5 and Table 6, respectively. The content of FA with more than 16 carbon atoms (>16) increased by 7 and 11% (*p* < 0.001) in the milk of the goats fed with the OC10 and OC20 diets, respectively, with concomitant decreases in de novo FA (<C16) secretion (*p* < 0.01). The same results were observed with the values of FA expressed as g/day. The total SFAs, as well as individual SFAs, like C4:0, C6:0, C14:0, C16:0, and C17:0, were decreased via the addition of OC in the goats’ diets. No diet effect was observed on the percentage of C18:0 among treatments. In contrast, the content of 18-C MUFAs, like C18:1 cis-9, as well as C18:1 trans-10, C18:1 trans-11, C18:1 trans-12, C18:1 cis-11, and C18:1 cis-13, were significantly elevated in the milk of the OC groups compared to the milk of the OC0 group (*p* < 0.001). Concerning the concentrations of other MUFAs, the C10:1 cis-9 content was reduced in OC10 and OC20, and the percentages of C16:1 cis-9 and C16:1 cis-7 were increased in OC20, while other monoenes were not affected in the milk of the OC groups.

There were no significant differences in the concentration of the total PUFAs among treatments, whereas the levels of CLA cis-9, trans-11 were enhanced by 11 and 21% in the OC10 and OC20 diets, respectively, compared to the OC0 group. Similar content of the CLA trans-10, cis-12, as well as other PUFAs, such as linoleic (C18:2n-6) and α-linolenic (C18:3n-3) acids, was observed among the treatments. Among the 20-C PUFAs, the content of C20:5n-3 and C22:5n-6 was reduced via the OC inclusion in the goats’ diets; in contrast, no diet effect on the concentrations of other 20-C acids was observed. The milk’s atherogenicity index diminished by 11% and 19% in the OC10 and OC20 treatments, respectively, compared to the control diet, while the different types of diets offered did not affect the desaturation index.

The differences that were observed in the concentrations of individual FAs were reflected in changes in the short, medium, and long-chain FAs (SCFAs, MCFAs, and LCFAs, respectively) expressed either as a % of the total FA or the FA yield per day. In particular, LCFAs were significantly increased, whereas MCFAs were reduced via the inclusion of 10 and 20% (DM) of OC in the goats’ diets (*p* < 0.001), while no statistically significant diet effect on the content of SCFAs among treatments was demonstrated (Table 5 and Table 6).

### 3.4. Gene Expression

**Figure 1:**
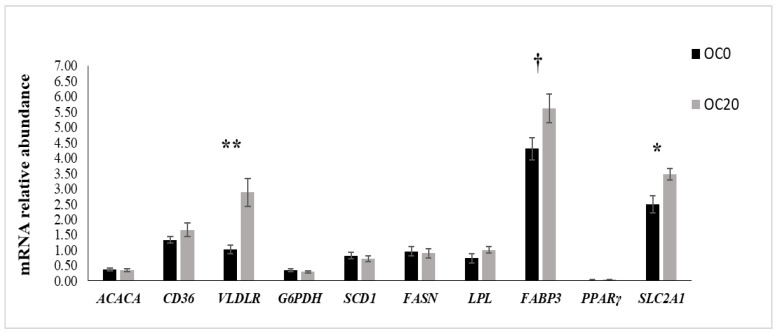
mRNA relative abundance (means ± SE) of lipogenic genes and transcription factors of the mammary tissue in dairy goats fed a control (OC0) diet (black bar) or the conventional diet supplemented with 20% (DM) of ensiled olive cake (OC20; gray bar). (mRNA levels are expressed as abundance relative to the geometric mean of *UXT*, *RPS9,* and *RPS15* mRNA). * *p* < 0.05; ** *p* < 0.01; ^†^ *p* < 0.1: tendency. *ACACA* = acetyl-CoA-carboxylase A; *FASN* = fatty acid synthase; *G6PDH* = glucose 6 phosphate dehydrogenase; *VLDLR* = very-low-density lipoprotein receptor; *LPL* = lipoprotein lipase; *SLC2A1* = solute carrier family 2, member 1; *CD36* = fatty acid translocase; *FABP3* = fatty acid binding protein 3; *PPARγ* = peroxisome proliferator-activated receptor γ; *SCD1* = stearoyl-CoA desaturase 1.

**Figure 2:**
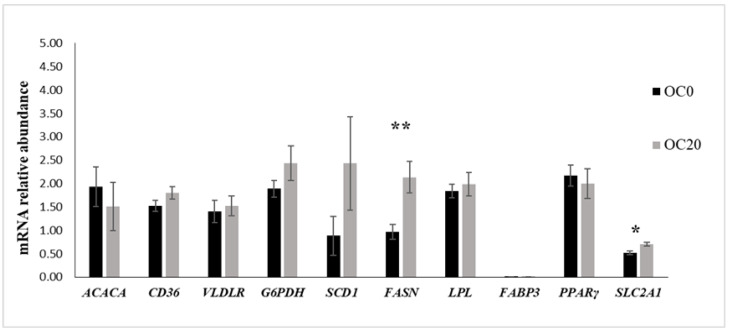
mRNA relative abundance (means ± SE) of lipogenic genes and transcription factors of the adipose tissue in dairy goats fed a control (OC0) diet (black bar) or the conventional diet supplemented with 20% (DM) of ensiled olive cake (OC20; gray bar). (mRNA levels are expressed as abundance relative to the geometric mean of UXT, RPS9 and RPS15 mRNA). * *p* < 0.05; ** *p* < 0.01. ACACA = acetyl-CoA-carboxylase A; FASN = fatty acid synthase; G6PDH = glucose 6 phosphate dehydrogenase; VLDLR = very-low-density lipoprotein receptor; LPL = lipoprotein lipase; SLC2A1 = solute carrier family 2 member 1; CD36 = fatty acid translocase; FABP3 = fatty acid binding protein 3; PPARγ = peroxisome proliferator-activated receptor γ; SCD1 = stearoyl-CoA desaturase 1.

Figure 1 and Figure 2 present the mRNA abundance of candidate genes studied in the mammary gland and adipose tissues, respectively. In the mammary tissues, the mRNA abundance of *VLDLR* (*p* < 0.01) and *SLC2A1* (*p* < 0.05) was increased, while the expression of *FABP3* tended to be elevated (*p* = 0.05) in goats fed with the OC20 diet. No diet effects were demonstrated on the mRNA abundance of any of the remaining genes examined in the mammary tissue. In the perirenal adipose tissue, the mRNA expression of *FASN* and *SLC2A1* was upregulated in goats fed 20% (DM) ensiled OC, while the expression of the other genes examined was unaffected.

## 4. Discussion

The present study showed that the milk yield was not affected by the 10 or 20% (DM) inclusion rates of ensiled OC, and it concurs with previous studies that have tested iso-nitrogenous and iso-energetic diets containing either 17% (DM) of ensiled OC [36] or 20% DM of ensiled by-products of both OC and olive leaves [14] in dairy goats. Similarly, studies conducted on dairy ewes showed that the milk yield was not affected by the inclusion of different forms of processed OC up to 20% of the diet DM [5,6,18,37,38] or, in cattle, when feeding ensiled or dried OC at rates up to 15% DM led to similar results [7,12,36,39,40].

The substitution of forage with 10 or 20% DM of ensiled OC in the goats’ diets significantly increased the milk fat content in the present study, similar to Hadjipanayiotou [36] and Arco-Pérez et al. [14], while the milk fat yield also tended to be elevated in the OC groups. Consistent with these findings in goats, a tendency of increased fat percentages and significantly elevated milk fat yields was reported in cows [7,12] when the animals were fed diets containing 10% dried or ensiled OC, respectively. Furthermore, significantly elevated milk fat percentages have been reported in dairy ewes by Hadjipanayiotou [36], who tested ensiled OC at a 15% DM inclusion rate, but not in our recent studies with inclusion rates of up to 20% DM [5,6].

The milk protein percentage was numerically increased through both the OC treatments but significantly only at the 20% inclusion of ensiled OC in the diet DM compared to the control. Increased milk protein content was observed in bovine milk following 10% (DM) of dried OC supplementation [12] but not with 10% (DM) of ensiled OC inclusion in cows [7]. It has been well stated that reducing the proportion of forage in ruminant diets increases the milk protein content and yield, and this effect is most likely attributed to the lower energy content of high-forage diets [41]. Although, in the present study, forage was replaced with OC silage, the concentrate ratio was kept at 60% DM for all diets, and the treatments were iso-energetic and isoproteic. Nevertheless, the in vitro study by Pallara et al. [42] examining the effect of OC on rumen microbial communities showed increased rumen volatile FA production, suggesting that OC inclusion could enhance rumen microbial activity and, consequently, the microbial protein supply and content in milk, but further research is needed to elucidate such a mechanism.

The ensiled OC inclusion positively affected milk FA composition by reducing SFAs and the atherogenicity index and increasing UFA in both the OC10 and OC20 groups. The content, as well as the g/day of SFAs with fewer than 16 carbons (C < 16), mainly MCFA, linearly decreased with an increasing OC proportion in the goats’ diets. These results are in line with previous studies on small ruminants [5,8,9,10,11,13,15] and cows [7,12] reporting reduced de novo FA percentages or g/day and increased levels of LCFA via feeding different diets containing various forms of processed OC. In contrast, Arco-Pérez et al. [14] reported increased SFAs and decreased UFA content in milk when 20% (DM) of goats’ diet was replaced with a mix of olive oil by-products, namely ensiled OC and olive leaves, and this discrepancy could be attributed to the different by-products added. It is known that the milk FAs of chain length C4 to C14 and approximately part of C16 are synthesized de novo in the mammary gland, whereas the remainder of C16 and LCFA present in milk are derived from the diet or body reserves [43]. Therefore, the reduction of C < 16 FAs expressed either as percentages or g/day observed in the present study could be due to either a higher secretion of LCFAs from the blood and/or a lower de novo synthesis of FAs in the mammary gland [43,44]. The de novo FA secretion in ruminant milk can be inhibited via bioactive FAs, including *trans* monoenes and CLAs, with the most well-documented bioactive FA being the CLA *trans*-10, *cis*-12 in cows [22]. Since this FA did not differ among the treatments in our study, other FA intermediates produced during the incomplete ruminal biohydrogenation (BH) of dietary LCFAs, such as C18:1 *trans*-10, suggested previously to play a similar role [22], were found to be elevated in the milk of the goats fed OC in our study. As indicated by Dorea and Armentano [44], this reduction in de novo synthesized lipids could occur without affecting the milk fat percentage, which was found to have increased in the OC groups in our study. Thus, summarizing the above data from milk fat percentages and the FA profile, either a higher level of dietary LCFAs from the blood and/or a decreased de novo synthesis of MCFAs via this specific *trans* isomer may be suggested as the underlying mechanism resulting in the altered milk FA composition observed for both OC treatments. However, the latter mechanism is not supported by our results on gene expression, as none of the tested genes involved in lipid metabolism were downregulated with the inclusion of 20% OC in the diet DM.

In the present study, the content and yield of total MUFAs were higher in the milk of the OC-supplemented groups compared to the control. Similar to our results, previous studies on small ruminants [5,8,9,10,11,13,15,45] and cows [7,12,40] have reported a linear decline in SFA content with concomitant increased levels of MUFAs via supplemented diets with various forms of processed OC. It can be assumed that dietary MUFAs, escaping rumen BH, were transferred to milk FA content through mammary uptake from the plasma, contributing to the higher MUFA content of the milk [20]. Furthermore, the inclusion of lipids rich in 18-C UFAs in dairy animal nutrition could increase *cis* and *trans* isomers of C18:1 and C18:2 that arise from ruminal the metabolism and mammary desaturation of C18:0 and C18:1 *trans* (mainly 7 and 11) [1]. Indeed, the linearly increased concentration of oleic acid found in the milk as a result of increasing amounts of OC in the diet could be due to either the action of Δ9-desaturase in the udder with the substrate being stearic acid or its direct transfer from feed [43]. However, considering the results of the Δ9-desaturation index, which did not differ among the groups, and the unaffected expression of the *SCD1* gene in the current trial, the elevated concentration of oleic acid and MUFAs is likely related more to the diet than to Δ9-desaturase activity.

The significantly increased levels of the CLA *cis*-9 *trans*-11 (rumenic acid: RA) observed in the milk fat of the goats fed the 10 or 20% (DM) ensiled OC diets in the present study have also been found in previous studies with goats [13,14,15] or ewes [5,6] and cattle that included OC at 10% DM [7,12], while contradictory results were observed in some studies that were implemented with sheep fed different forms of processed OC [9,10]. It is known that RA can originate from two pathways; it can be synthesized either via the action of Δ9-desaturase in the mammary gland with vaccenic acid as the substrate, which is responsible for about 60% of RA secretion in milk, or through the BH of UFAs via rumen bacteria [43].

In order to further investigate the molecular mechanisms contributing to the observed effects due to ensiled OC supplementation, the expression of selected genes involved in lipid metabolism on the mammary and adipose tissues of goats fed the OC20 and the OC0 diets was examined. The results showed that the decreased MCFAs (medium-chain FAs) derived from de novo lipogenesis (expressed as percentages of FAs or FA g per day) of the OC treatment were not accompanied by changes in the mRNA abundance of the selected genes studied in the present study and involved in mammary lipid synthesis. These results are in line with studies reporting no effect on the caprine and/or bovine [24,46,47,48,49,50] or ovine [51,52] mammary levels of the expression of *ACACA*, *FASN*, and *SCD1* as an effect of dietary lipid supplementation. Similarly, the results of our previous work with OC feeding in cows [7] showed that a decreased proportion of MCFAs in OC milk could occur with no changes in the expression of genes involved in mammary lipid synthesis.

In the present study, the increased LCFA content and yield in the milk of the OC20 group coincided with the significantly elevated expression of *VLDLR* and *SLC2A1* in the mammary glands of the goats. *SLC2A1* is the predominant transporter of LCFAs into cells via a saturable protein-mediated mechanism, while VLDLR, in combination with LPL, takes up and hydrolyzes triacylglycerides [53]. In our previous work [7], elevated secretion of 18-C FAs was observed in the milk of cows fed a diet containing 10% (DM) ensiled OC, while the bovine mammary levels of the expression of *VLDLR* and *SLC2A1* remained unaffected, suggesting that these genes might be regulated at other levels, such as post-transcriptional or post-translational levels. Most nutrigenomic studies of goats [24,46,48] reported no significant effect on the expression of any of the genes involved in mammary lipid metabolism after dietary supplementation with lipids. These discrepancies could be attributed to the different natures and doses of lipid supplements or species/breed specificity. The overexpression of *SLC2A1* and *VLDLR* observed in the mammary glands of the OC20 group in the current study could be attributed to the higher amounts of LCFAs, particularly oleic and stearic acid, in the OC diet compared to the control that were thereafter secreted in the milk. Also, a tendency for a higher mammary mRNA abundance of *FABP3* with the OC20 diet was demonstrated in the present study. This finding is consistent with that of Invernizzi et al. [54], who reported a significant increase in *FABP3* expression in the mammary gland of lactating cows fed marine-originated lipids and in which milk fat depression (MFD) was induced as a result. However, MFD was not observed in the present study, and other nutrigenomic studies in goats [24,46,48] and ewes [51] have shown no effect on the mammary mRNA abundance of *FABP3* as an effect of lipid supplementation. As indicated, FABP3 plays an important role during lactation in cows’ [53] and goats’ [55] lipid metabolism, channeling palmitic and stearic acids for desaturation as it provides stearoyl-CoA to SCD. This suggests that *FABP3* is a master regulator in the milk fat synthesis signaling pathway, in cooperation with FAs [56]. Indeed, the in vitro study by Liang et al. [56] reported that the addition of stearic and palmitic acids to cows’ mammary gland epithelial cell cultures generated an increase in *FABP3* abundance. In the present study, the increased mRNA abundance of *FABP3* could be related to the higher content of C18:0 contained in the OC20 diet compared to the control.

The mRNA abundance of *FASN* was significantly increased in the adipose tissue of the animals that received the OC20 diet in the present study. Previous studies testing plant oils in small ruminants [48,49,52] or in cow [57] diets reported no significant effects on the *FASN* mRNA abundance or on the expression of any of the genes tested in the adipose tissue. A tendency of increased *FASN* mRNA abundance in the adipose tissue has been observed in cows after plant oil supplementation [58]. At the same time, in another study [59], the intravenous infusion of CLA *trans*-10, *cis*-12, which is an inhibitor of de novo milk FA synthesis in the mammary tissue, upregulated the FASN in the adipose tissue of dairy cows. However, in that study, MFD was observed due to CLA *trans*-10, *cis*-12 infusion, resulting in a redirection of nutrient distribution towards the adipose tissue in favor of fat store reconstitution, providing a possible explanation of the FASN increment. In contrast, MFD was not observed in the present study, and moreover, the milk fat yield tended to be increased in the OC-fed groups. Furthermore, the CLA *trans*-10, *cis*-12 isomer has been found to increase the expression of adipogenic *FASN* and other lipid synthesis enzymes in cows [22], but in dairy goats, adipogenesis is unresponsive or less sensitive to CLA *trans*-10, *cis*-12 [43], suggesting that other intermediates could have influenced the expression of FASN in the adipose tissues of the present study. Nevertheless, an in vitro study examining the effects of different LCFAs on the adipogenesis of bovine adipocytes reported an increase in FASN expression for some LCFAs, including oleic acid, and those effects were dose-dependent [60]. It is possible that the increment of specific LCFAs, for example, oleic acid, C18:1 *trans*-10, or other monoenes, arising from oleic acid isomerization may be associated with the upregulation of *FASN* in the adipose tissues of lactating goats. Nevertheless, further studies are required to elucidate the specific role of oleic acid or other LCFA derivatives on lipid metabolism when feedstuffs rich in oleic acid are offered to dairy goats.

In the present study, the OC20 diet upregulated the *SLC2A1* in the adipose tissue of the goats. This was not observed in our previous work, in which lactating cows were fed with 10% (DM) ensiled OC [7], possibly due to species-specific differences or increased OC diet content in the case of goats. The *SLC2A1*-gene encoding glucose transporter 1(*GLUT1*) protein is a key transporter of glucose into the cells [61]. The supplementation with 20% ensiled OC led to enhanced *SLC2A1* expression, which could likely increase LCFA and glucose uptake in the adipose tissue of goats. Overall, our findings suggest that the inclusion of ensiled OC enhances FA synthesis and transport via the increased expression of *FASN* and *SLC2A1* in caprine perirenal fat. Moreover, the results of this study suggest that mammary and adipose tissues’ lipogenic pathways are at least in part involved in the response of mammary lipid secretion due to the dietary addition of ensiled OC.

## 5. Conclusions

As indicated in the present study, OC inclusion rates of 10 and 20% in goats’ diets improved milk fat quality by reducing SFAs and increasing unsaturated FA percentages, including oleic, vaccenic, and rumenic, which are all related to positive effects on human health. Supplementation with 20% ensiled OC led to the upregulation of genes involved in FA uptake and translocation in the mammary gland, consistent with increased LCFA levels. Reduced de novo synthesis in the mammary gland was accompanied by the upregulation of genes involved in FA and glucose transport and FA synthesis in the adipose tissue. These results suggest that the inclusion of ensiled OC at rates of 20% in DM is a useful additive for dairy goats’ diets that can improve the fat and protein content, as well as the FA profile, of milk and possibly other related dairy products without adverse effects on milk production.

## Figures and Tables

**Table 1:** Ingredients and chemical composition of diets containing 0 (control, OC0), 10 (OC10), and 20 (OC20) g of ensiled olive cake per 100 g DM and olive cake silage used.

	Treatment			
Item	OC0	OC10	OC20	OC Silage
Ingredient composition, %				
OC	-	9.7	19.8	
Alfalfa	11.1	10.9	10.3	
Barley hay	17.4	12.8	8.0	
Barley straw	10.8	6.2	1.7	
Concentrate mix	60.7	60.5	60.2	
Chemical composition, % DM				
Dry matter, %	88.60	82.96	78.98	47.74
Crude protein, % DM	15.86	15.89	15.92	5.32
Crude fat, % DM	2.30	3.07	3.82	6.88
Crude fiber, % DM	18.20	18.75	19.30	49.53
Ash, % DM	7.13	6.90	6.67	2.71
Ca	1.00	0.99	0.98	-
P	0.39	0.39	0.38	-
Na	0.24	0.23	0.23	-
aNDF, % DM	37.44	37.71	38.01	71.56
ADF, % DM	22.89	23.06	23.25	54.93
Metabolized Energy (MJ/kg)^2^	10.65	10.51	10.37	-

**Table 2:** Primers’ sequences (5′ to 3′) and efficiencies used in real-time quantification (q)PCR.

Symbol	Name	Forward (F) and Reverse (R)	Access Number	Amplicon (bp)	R^2^	Efficiency (%)
*ACACA*	Acetyl-Coa-Carboxylase A	F: ATCATCACCATCAGCCTGGTTA R: AGGTGTATACTTCCCTCCCGA	XM_018064168.1	154	0.992	93
*FASN*	Fatty Acid Synthase	F: CTCCATCCTCGCTCTCCTTC R: CATATAGTCCCGCCTTCCACC	NM_001285629.1	200	0.997	87
*G6PDH*	Glucose 6 Phosphate Dehydrogenase	F: CTTCCATCAGGCCGATACGC R: GGGTAGCTTTGAAGAAGGGCTC	XM_018044339.1	200	0.997	94
*VLDLR*	Very-Low-Density Lipoprotein Receptor	F: CTGCTGTGGAAATGCGATGG R: TCTCATATGGCACTGTTCTGGG	XM_018052033.1	192	0.993	89
*LPL*	Lipoprotein Lipase	F: CAACAAGGTCAGAGCCAAAAGA R: ACTTCAGGCAGGGTAAAAGGG	NM_001285607.1	198	0.998	88
*SLC2A1*	Solute Carrier Family 2 Member1	F: GTCGTGTCGCTGTTTGTGG R: GCCTGGACCCACTTCGAAAA	NM_001314223.1	189	0.991	90
*FAT/CD36*	Fatty Acid Translocase	F: ATTGACACATACAAAGGCAGAAAGAAT R: AGCTCCGAACACAGCATAGAT	XM_018046617.1	176	0.998	99
*FABP3*	Fatty Acid Binding Protein	F: CGAGTTCGATGAGACCACGG R: CATGGGTGAGTGTCAGAATGAGT	NM_001285701.1	155	0.993	99
*PPARγ*	Peroxisome Proliferator Activated Receptor γ	F: AAGCGTCAGGGTTCCACTATG R: CCGAACCTGATGGCGTTATGA	NM_001285658.1	199	0.997	96
*SCD1*	Stearoyl-Coa Desaturase	F: ACATTGATCCCCACCTGCAA R: TCAAAAACGTCATTCTGGAACGC	NM_001285619.1	185	0.998	94
Housekeeping genes						
*UXT*	Ubiquitously Expressed Transcript	F: CGTAAGAGCAATCTCCTCACAGA R: TGTAGCTCTCTAAGCCCCTCTA	XM_005700842.2	104	0.997	96
*RPS9*	Ribosomal Protein S9	F: AAGCTGATCGGCGAGTACG R: TTCATCTTGCCCTCGTCCAG	XM_018063497.1	191	0.998	98
*RPS15*	Ribosomal Protein S15	F: GCATTGAGACCCCGCGATAA R: TTCTACTTCCGCCATCTTGCC	XM_018050438.1	171	0.990	88

**Table 3:** Fatty acid composition (expressed as a percentage of the total fatty acid methyl esters) of the dietary treatments containing 0 (control, OC0), 10 (OC10), or 20 (OC20) g of ensiled olive cake per 100 g DM and olive cake silage (OC).

Item	Treatments					
	OC0	OC10	OC20	SEM	*p*-Value	Ensiled OC
C14:0	1.19 ^a^	0.67 ^b^	0.41 ^c^	0.14	**	-
C16:0	37.29 ^a^	30.18 ^b^	29.61 ^b^	0.79	**	13.45
C16:1 *cis*-9	0.35 ^c^	0.62 ^b^	0.97 ^a^	0.05	**	1.48
C18:0	1.78 ^c^	2.46 ^b^	3.54 ^a^	0.10	***	4.15
C18:1 *cis*-9	21.00 ^b^	34.82 ^a^	37.00 ^a^	2.70	***	63.58
C18:2n-6	25.52 ^a^	19.97 ^b^	18.30 ^c^	0.50	**	13.12
C18:3n-3	2.42 ^a^	2.38 ^b^	2.12 ^b^	0.15	*	1.46

^a–c^ The means within a row not sharing a common superscript differed due to the different diets examined * *p* < 0.05, ** *p* < 0.01, *** *p* < 0.001.

**Table 4:** Dry matter intake (DMI), milk production, and chemical composition of milk from goats fed diets containing 0 (control, OC0), 10 (OC10), or 20 (OC20) g of ensiled olive cake per 100 g DM.

	Treatment				*p*-Value ^1^		
Item	OC0	OC10	OC20	SEM	D	T	D × T
DMI (kg/d)	2.53	2.57	2.63	0.03	NS	NS	NS
Milk (kg/d)	2.72	2.79	2.83	0.10	NS	NS	NS
FCM ^2^ (kg/d)	2.35	2.43	2.50	0.07	NS	**	NS
Fat (%)	3.08 ^c^	3.20 ^b^	3.30 ^a^	0.06	*	***	NS
Fat (kg/d)	0.082	0.085	0.091	0.004	^†^	*	NS
Protein (%)	3.68 ^b^	3.74 ^b^	3.85 ^a^	0.02	***	***	NS
Protein (kg/d)	0.106	0.111	0.114	0.004	NS	^†^	NS
Lactose (%)	5.33	5.61	5.48	0.10	NS	NS	NS
SNF (%)	9.56	10.07	10.05	0.08	NS	NS	NS

^a–c^ The means within a row not sharing a common superscript differed due to the different diets examined (*p* < 0.05); ^1^ probability of significant effects due to the diet (D), time (T), and their interaction (D × T); * *p* < 0.05; ** *p* < 0.01; *** *p* < 0.001; NS: non-significant; ^†^ *p* < 0.1: tendency, ^2^ 4% fat-corrected milk (FCM): milk yield × (0.411 + 0.147 × fat).

**Table 5:** Fatty acid composition (expressed as a percentage of the total fatty acid methyl esters) of milk from goats fed diets containing 0 (control, OC0), 10 (OC10), or 20 (OC20) g of ensiled olive cake per 100 g DM.

	Treatment				*p*-Value ^1^		
Item	OC0	OC10	OC20	SEM	D	T	D × T
C4:0	0.80 ^a^	0.72 ^b^	0.75 ^ab^	0.01	*	**	^†^
C5:0	0.12	0.15	0.13	0.001	NS	NS	NS
C6:0	1.53 ^a^	1.37 ^b^	1.45 ^ab^	0.04	**	NS	**
C7:0	0.36 ^b^	0.45 ^a^	0.38 ^ab^	0.003	NS	NS	NS
C8:0	2.32 ^a^	2.13 ^b^	2.15 ^b^	0.05	**	*	**
Octanoic	0.039 ^a^	0.037 ^a^	0.028 ^b^	0.002	***	NS	NS
C9:0	0.097	0.117	0.096	0.007	^†^	NS	NS
C10:0	5.79 ^b^	6.69 ^a^	5.37 ^b^	0.28	**	**	*
C10:1 *cis*-9	0.21 ^a^	0.16 ^b^	0.17 ^b^	0.008	***	***	NS
C11:0	0.15 ^ab^	0.19 ^a^	0.14 ^b^	0.01	*	NS	^†^
C12:0	4.09	4.06	3.76	0.12	^†^	**	*
C12:1 *cis*-9	0.038	0.032	0.031	0.003	NS	***	NS
C13:0 iso	0.02	0.02	0.01	0.002	NS	NS	NS
C13:0	0.19	0.21	0.18	0.01	NS	**	NS
C14:0	9.00 ^a^	8.47 ^b^	8.05 ^b^	0.15	***	***	***
C14:0 *iso*	0.07 ^a^	0.051 ^b^	0.05 ^b^	0.003	***	NS	*
C14:1 *cis*-9	0.44	0.15	0.15	0.12	NS	NS	NS
C15:0	1.24 ^a^	1.23 ^a^	1.13 ^b^	0.03	*	**	*
C15:0 *iso*	0.18 ^a^	0.14 ^b^	0.13 ^b^	0.005	***	**	***
C15:0 antiso	0.43 ^a^	0.36 ^b^	0.34 ^b^	0.011	***	***	***
C16:0	25.39 ^a^	22.89 ^b^	22.37 ^b^	0.38	***	^†^	NS
C16:0 *iso*	0.31 ^a^	0.26 ^b^	0.27 ^b^	0.008	***	**	***
C16:1 *cis*-9	0.13 ^b^	0.15 ^b^	0.18 ^a^	0.008	***	NS	NS
C16:1 *cis*-7	0.28 ^b^	0.27 ^b^	0.31 ^a^	0.009	**	**	NS
C17:0	1.07 ^a^	1.01 ^b^	0.94 ^c^	0.02	***	***	***
C17:0 *iso*	0.54 ^a^	0.46 ^b^	0.41 ^b^	0.01	***	**	***
C17:0 antiso	1.43	1.38	1.37	0.02	NS	NS	**
C17:1 *cis*-9	0.34	0.33	0.32	0.01	NS	***	NS
C18:0	10.40	10.12	10.35	0.46	NS	^†^	NS
C18:0 *iso*	0.094	0.093	0.090	0.004	NS	***	**
C18:1 *trans*-10	0.31 ^b^	0.37 ^ab^	0.39 ^a^	0.01	***	***	NS
C18:1 *trans*-11	4.75 ^b^	5.70 ^a^	6.12 ^a^	0.26	***	NS	^†^
C18:1 *trans*-12	0.33 ^c^	0.41 ^b^	0.68 ^a^	0.05	***	^†^	NS
C18:1 *cis*-9	18.48 ^c^	21.12 ^b^	23.05 ^a^	0.49	***	NS	NS
C18:1 *cis*-11	0.71 ^b^	0.86 ^a^	0.91 ^a^	0.02	***	^†^	NS
C18:1 *cis*-12	0.30	0.30	0.31	0.01	NS	NS	***
C18:1 *cis*-13	0.17 ^b^	0.21 ^a^	0.23 ^a^	0.01	***	***	NS
C18:1 *cis*-16	0.26	0.29	0.28	0.02	NS	^†^	NS
C18:1 *trans*-16	0.27	0.25	0.24	0.01	NS	NS	***
C18:2 *cis*-9, *trans*-13/*trans*-8, *cis*-12	0.21	0.21	0.21	0.006	NS	**	NS
C18:2 *trans-9*, *cis*-13/*trans*-8, *cis*-12	0.13	0.14	0.15	0.005	^†^	**	NS
C18:2 *trans*-11, *cis*-15	0.11	0.13	0.13	0.008	NS	NS	NS
C18:2n-6	4.58	4.59	4.69	0.18	NS	^†^	NS
C18:3n-6	0.04	0.04	0.04	0.002	NS	**	NS
C18:3n-3	0.35	0.36	0.38	0.01	NS	^†^	NS
C19:1 *cis*-9	0.043	0.043	0.042	0.003	NS	***	NS
C20:0	0.25 ^b^	0.25 ^b^	0.27 ^a^	0.006	**	***	NS
C21:0	0.066 ^a^	0.055 ^b^	0.061 ^ab^	0.002	***	**	**
C22:0	0.074	0.068	0.069	0.002	NS	***	*
C23:0	0.031	0.022	0.020	0.002	***	NS	NS
C24:0	0.014 ^ab^	0.015 ^a^	0.012 ^b^	0.001	**	NS	NS
CLA *cis*-9, *trans*-11	0.41 ^c^	0.46 ^b^	0.52 ^a^	0.01	***	^†^	*
CLA *trans*-10, *cis*-12	0.062	0.068	0.073	0.005	NS	NS	NS
CLA *trans, trans*	0.11	0.15	0.15	0.01	^†^	^†^	NS
C20:2n-6	0.028	0.032	0.025	0.004	NS	NS	NS
C20:3n-6	0.04	0.03	0.03	0.01	NS	^†^	NS
C20:3n-3	0.02	0.02	0.02	0.001	NS	***	NS
C20:4n-6	0.26	0.24	0.24	0.009	NS	***	NS
C20:5n-3	0.025 ^a^	0.018 ^b^	0.016 ^b^	0.001	***	NS	NS
C22:4n-6	0.019	0.016	0.016	0.001	NS	***	**
C22:5n-6	0.043 ^a^	0.032 ^b^	0.033 ^b^	0.001	***	***	^†^
C22:5n-3	0.055 ^a^	0.046 ^ab^	0.042 ^b^	0.002	**	***	NS
SCFA ^2^	10.93	11.09	10.22	0.30	NS	^†^	*
MCFA ^3^	41.49 ^a^	38.30 ^b^	36.99 ^b^	0.52	***	***	**
LCFA ^4^	46.33 ^c^	49.92 ^b^	52.39 ^a^	0.69	***	NS	NS
<C16	26.94 ^a^	26.62 ^a^	24.73 ^b^	0.46	**	NS	**
>C16	46.55 ^b^	50.19 ^a^	52.40 ^a^	0.69	***	NS	ND
SFA	65.51 ^a^	62.46 ^b^	59.68 ^c^	0.69	***	NS	NS
MUFA	29.92 ^b^	30.88 ^ab^	32.98 ^a^	0.64	**	***	***
PUFA	6.44	6.43	6.74	0.22	NS	NS	NS
Atherogenicity index ^5^	1.16 ^a^	1.03 ^b^	0.92 ^b^	0.03	***	^†^	NS
Desaturation index ^6^	1.76	1.72	1.85	0.07	NS	***	NS

^a–c^ The means within a row not sharing a common superscript differed due to the different diets examined (*p* < 0.05); ^1^ probability of significant effects due to diet (D), time (T), and their interaction (D × T); * *p* < 0.05; ** *p* < 0.01; *** *p* < 0.001; ^†^ *p* < 0.1: tendency; NS: non-significant; ^2^ SCFA: short-chain fatty acids (C4:0 to C8:0); ^3^ MCFA: medium-chain fatty acids (C10:0 to C16:1); ^4^ LCFA: long-chain fatty acids (C17:0 and above); ^5^ atherogenicity index = (C12:0+ 4 × C14:0 + C16:0)/(ΣMUFA + ΣPUFA); ^6^ desaturation index = (C14:1 *cis*-9/C14:0 + C14:1 *cis*-9) × 100.

**Table 6:** Milk’s fatty acid yield (g/d) in goats fed diets containing O (control, OC), 10 (OC10), or 20 (OC20) g of ensiled olive cake (the selected group of fatty acids are presented).

Item	Treatment			SEM	*p*-Value ^1^		
	OC0	OC10	OC20		D	T	D × T
SCFA ^2^	9.56 ^ab^	10.50 ^a^	7.94 ^b^	0.55	NS	*	NS
MCFA ^3^	37.02 ^a^	35.02 ^b^	30.11 ^c^	1.72	***	***	NS
LCFA ^4^	38.10 ^b^	42.92 ^a^	44.44 ^a^	2.10	**	***	NS
<C16	25.47 ^a^	24.58 ^a^	20.52 ^b^	1.58	**	NS	NS
>C16	38.64 ^c^	41.96 ^b^	46.83 ^a^	2.19	**	NS	NS
SFA	59.52 ^a^	56.14 ^b^	50.34 ^c^	1.69	**	**	NS
MUFA	25.81 ^b^	28.33 ^ab^	29.24 ^a^	1.64	***	NS	NS
PUFA	5.50	5.44	5.97	0.22	NS	***	NS

^a–c^ The means within a row not sharing a common superscript differed due to the different diets examined (*p* < 0.05); ^1^ probability of significant effects due to diet (D), time (T), and their interaction (D × T); * *p* < 0.05; ** *p* < 0.01; *** *p* < 0.001; NS: non-significant; ^2^ SCFA: short-chain fatty acids (C4:0 to C8:0); ^3^ MCFA: medium-chain fatty acids (C10:0 to C16:1); ^4^ LCFA: long-chain fatty acids (C17:0 and above).

## Data Availability

All data presented in this paper are original to this study. The data used to support the findings of this study are available from the corresponding author upon request.
